# Immune Checkpoint Inhibitors and Survival Disparities by Health Insurance Coverage Among Patients With Metastatic Cancer

**DOI:** 10.1001/jamanetworkopen.2025.19274

**Published:** 2025-07-07

**Authors:** Jingxuan Zhao, Ilana Graetz, David Howard, K. Robin Yabroff, Joseph Lipscomb

**Affiliations:** 1 Department of Surveillance and Health Equity Science, American Cancer Society, Atlanta, Georgia; 2 Rollins School of Public Health, Emory University, Atlanta, Georgia; 3 Winship Cancer Institute of Emory University, Atlanta, Georgia

## Abstract

**Importance:**

The introduction of immune checkpoint inhibitors (ICIs) has been associated with substantial improvement in median survival among individuals with cancer; however, people without health insurance coverage may be unable to afford ICIs due to their high costs.

**Objective:**

To examine the association between the introduction of ICIs and changes in survival disparities by health insurance coverage among people with a new diagnosis of advanced-stage cancer.

**Design, Setting, and Participants:**

This cross-sectional study was conducted among individuals who received a new diagnosis at age 18 to 64 years of stage IV melanoma (n = 12 048), non–small cell lung cancer (NSCLC; n = 152 610), or renal cell carcinoma (RCC; n = 18 782) who were identified from the National Cancer Database before and after US Food and Drug Administration approval of ICIs (January 1, 2002, to December 31, 2019, for melanoma and January 1, 2010, to December 31, 2019, for other cancers). Statistical analysis was conducted from December 2023 to April 2025.

**Exposure:**

Health insurance coverage at the time of diagnosis (private, Medicaid, or uninsured).

**Main Outcomes and Measures:**

The primary study outcome was 2-year overall survival. For each type of cancer, a 6-group propensity score weighting difference-in-differences (DID) approach was applied to examine the changes in 2-year survival before and after the ICI approval date among individuals without insurance or with Medicaid compared with those with private insurance.

**Results:**

Among the 183 440 individuals included, the mean (SD) age was 55.5 (7.0) years, and 56.5% were male. Two-year overall survival rates increased post-ICI approval among uninsured individuals (from 16.2% to 28.3%) and individuals with private insurance (from 28.7% to 46.0%) for those with a diagnosis of melanoma, resulting in a widening disparity of 6.1 percentage points (pp) (95% CI, 1.7-10.6 pp) after adjusting for sociodemographic characteristics. Similarly, among people with a diagnosis of NSCLC, the survival disparity between people without insurance and people with private insurance widened to 1.3 pp (95% CI, 0.2-2.3 pp). Survival differences between people with Medicaid and those with private insurance did not change significantly with the introduction of ICIs (DID for melanoma, −1.9 pp [95% CI, −5.6 to 1.8 pp]; DID for NSCLC, 0.4 pp [95% CI, −0.4 to 1.2 pp]; and DID for RCC, −3.8 pp [95% CI, −9.4 to 1.9 pp]).

**Conclusions and Relevance:**

This serial cross-sectional study found that the introduction of ICIs was associated with widening survival disparity between people without health insurance and those with private insurance. Policies expanding access to health insurance coverage options and making new treatments more affordable are needed.

## Key Points

**Question:**

Was the introduction of immune checkpoint inhibitors (ICIs) associated with increased disparities in survival by health insurance coverage among individuals with a new diagnosis of advanced-stage cancer?

**Findings:**

This serial cross-sectional study using nationwide cancer registry data found that after the introduction of ICIs, there was a statistically significant widening of the gap in survival outcomes between privately insured and uninsured individuals with a new diagnosis of stage IV melanoma, non–small cell lung cancer, or renal cell carcinoma.

**Meaning:**

This study suggests that programs aimed at improving health insurance coverage and providing comprehensive financial assistance to people without coverage may help to mitigate disparities in survival among individuals with a new diagnosis of advanced-stage cancer.

## Teaser

This cross-sectional study uses nationwide cancer registry data to examine the association between the introduction of immune checkpoint inhibitors and survival disparities by health insurance coverage among people with a new diagnosis of advanced-stage cancer.

## Introduction

First approved by the US Food and Drug Administration (FDA) in 2011 for treating stage IV melanoma, immune checkpoint inhibitors (ICIs)—primarily including anti-cytotoxic T-lymphocyte–associated protein 4 (eg, ipilimumab), anti-programmed cell death 1 or programmed cell death protein (eg, pembrolizumab and nivolumab), and anti-programmed cell death ligand 1 (eg, atezolizumab and durvalumab) therapies^1^—are now approved for treating multiple cancers, including melanoma, non–small cell lung cancer (NSCLC), and renal cell carcinoma (RCC).^2,3^ Compared with conventional therapies, ICIs have been shown to significantly improve median overall survival among patients across several cancer types in clinical trials, including metastatic melanoma (ipilimumab, 10.0 months vs 6.4 months), NSCLC (nivolumab, 12.2 months vs 9.4 months), and RCC (nivolumab, 25.0 months vs 19.6 months).^4,5,6^

In the US, disparities in cancer outcomes by health insurance coverage and socioeconomic status have persisted,^7^ and there are concerns that the introduction of advanced therapies, such as ICIs, could widen these disparities.^8^ Although ICIs are associated with substantial improvement in survival,^9,10^ people with lower incomes or without health insurance coverage may be unable to afford them due to their high costs.^8^ Before the introduction of ICIs, conventional treatments, such as radiotherapy and chemotherapy, were often used to treat people with a diagnosis of advanced-stage cancers. Immune checkpoint inhibitors are significantly more costly than conventional treatments, with a mean Medicaid reimbursed price per prescription starting at $7000 to $8000.^11^ The duration of ICI treatment ranges from a few months to several years,^11^ and the cumulative cost of ICI treatment alone for the entire regimen over time (based on list price) could be as high as $250 000.^12^

In the US, having health insurance is critical for access to care and is associated with better health outcomes.^13,14,15^ Because individuals without health insurance coverage may not be able to afford high-cost ICIs, they may not be able to benefit equitably from their introduction as a treatment option. However, no prior studies have directly examined changes in survival disparities by health insurance coverage associated with the introduction of ICIs. In this study, we used nationwide cancer registry data to examine the association of the introduction of ICIs with changes in survival by health insurance coverage among people with a new diagnosis of stage IV melanoma, NSCLC, or RCC.

## Methods

### Data and Sample

Data were obtained from the National Cancer Database (NCDB), a nationwide hospital-based registry that includes approximately 70% of all newly diagnosed cancer cases in the US from more than 1500 facilities accredited by the American College of Surgeons’ Commission on Cancer.^16^ The NCDB is jointly sponsored by the American Cancer Society and the American College of Surgeons. The NCDB contains individual-level data on demographic characteristics, tumor characteristics, health insurance coverage, and vital status. This serial cross-sectional study of secondary data was exempted by the Morehouse College institutional review board and patient consent was waived because the data are deidentified. The results reported here follow the Strengthening the Reporting of Observational Studies in Epidemiology (STROBE) reporting guideline.

We included individuals aged 18 to 64 years with a new diagnosis of stage IV cancers eligible for ICI treatment that was approved by the FDA before 2016, including melanoma (first approved: ipilimumab, on March 25, 2011), NSCLC (first approved: nivolumab, on March 4, 2015), and RCC (first approved: nivolumab, on November 23, 2015; eTable 1 in Supplement 1).^17,18^ To create balanced samples before and after the approval of ICIs for each cancer site, we identified individuals aged 18 to 64 years who received a diagnosis of stage IV melanoma between January 1, 2002, and December 31, 2019, and a diagnosis of stage IV NSCLC or stage IV RCC between January 1, 2010, and December 31, 2019. Those who received a diagnosis at 65 years or age or older were excluded because they were eligible by age for Medicare coverage and less than 2% were uninsured.^19^ We did not include individuals who received a diagnosis in 2020 or 2021 because the COVID-19 pandemic disrupted health care delivery.^20^

This study focused specifically on comparing outcomes between individuals with private health insurance and those with Medicaid or those who were uninsured at diagnosis. We excluded individuals aged 18 to 64 years with Medicare or dual Medicare with Medicaid because their eligibility is based on disability or certain health conditions that may be associated with treatment options and survival outcomes. We excluded individuals with missing information on sex or with recorded sex other than male or female due to small sample size (n = 79). Individuals whose diagnosis date matched the date of death or last contact (n = 11 161) or with missing information on follow-up time or vital status (n = 1590) were also excluded (eFigure 1 in Supplement 1). We also compared the percentages of excluded individuals due to missing data across different health insurance groups and found that individuals with Medicaid coverage or those who were uninsured had a higher likelihood of exclusion due to missing data (eTable 2 in Supplement 1).

### Measures

All individual-level variables were derived from the Facility Oncology Registry Data Standards.^21^ Consistent with previous studies,^7,15,22^ health insurance at the time of cancer diagnosis was grouped into private (managed care, health maintenance organization, preferred provider organization, fee for service, TRICARE, military care, and insurance not otherwise specified); Medicaid (Medicaid and Medicaid administered through a managed care plan); and uninsured (not insured or not insured self-pay).

The primary study outcome was 2-year overall survival, which was treated as a time-to-event outcome and measured from date of diagnosis to date of death. Individuals were right censored in survival analyses if alive at the date of last contact, at the date when they turned 65 years of age (because most adults become eligible for Medicare coverage at 65 years of age), at the end of 2-year follow-up, or at the end of study follow-up (December 31, 2019). Individuals who received a diagnosis before ICI approval were right censored on the date of the ICI approval to exclude the potential association of subsequent ICI treatment with their survival. We included all diagnosis dates within the study period to ensure data comprehensiveness, although not all individuals had a full 2-year follow-up, which is a commonly used approach in survival analysis.^23,24^

Because the mortality data in the NCDB are all-cause only (ie, cause of death is not available), to examine if all-cause mortality is a reasonable proxy for cancer-specific mortality, we used data from 22 cancer registries from the Surveillance, Epidemiology, and End Results (SEER) program to compare differences in overall survival and cancer-specific survival for individuals aged 18 to 64 years who received a diagnosis of stage IV cancer of study interest over the same time period (data obtained from SEER*Stat, version 8.4.2). Overall survival and cancer-specific survival rates were generally similar for all selected cancers (eTable 3 in Supplement 1).

### Statistical Analysis

Statistical analysis was conducted from December 2023 to April 2025. Descriptive statistics were used to characterize the study sample by health insurance coverage. Kaplan-Meier survival curves were generated by pre-ICI and post-ICI approval periods and by health insurance coverage status (ie, privately insured, Medicaid, or uninsured) to visualize changes in survival.

For each cancer site, we used a difference-in-differences (DID) approach to examine the changes in 2-year overall survival before and after the FDA approval of ICIs among individuals with Medicaid or without health insurance compared with those with private health insurance. To account for potential bias introduced by changes in sample composition by health insurance coverage due to policies such as Medicaid expansion under the Patient Protection and Affordable Care Act (ACA), we used a standardized propensity score weighting approach in the DID models. A more detailed description of the analytic approach can be found in the eMethods in Supplement 1.^25,26^ Because ICIs were approved by the FDA as second-line treatment for NSCLC and RCC and because there might be lags between FDA approval and ICI uptake, we also conducted analyses excluding individuals who received a diagnosis in the first year after ICI approval for these cancers to account for the potential lag effects.

Weighted flexible parametric survival models were used to calculate the DID for 2-year overall survival, with health insurance coverage, before and after ICI approval, and their interaction term (health insurance coverage by pre- and post-ICI approval) as the main independent variables.^27,28^ Compared with the semiparametric Cox proportional hazards regression models, the flexible parametric survival models allow for direct estimation of the survival rates and differences in survival rates at a given follow-up time and are not constrained by the proportional hazards assumption^27^; consequently, such parametric models have been used in DID settings to analyze health policy changes and time-to-event outcomes.^23,24^ Included in these adjusted models were age group, sex, race and ethnicity (ascertained for the NCDB from medical records), Charlson-Deyo Comorbidity Index, zip code–level median household income as a percentage of the federal poverty level, rural-urban status, facility type, state of residence, and year of diagnosis. These variables were included as they were presumed, a priori, to be possible confounders of the analyses.

Adopting a commonly used approach,^23,24^ we tested the parallel trend assumption for the weighted DID models by grouping the pre-ICI approval data into quarterly intervals and examining the statistical significance of the health insurance-by-quarter interaction term using the adjusted flexible parametric survival models. Results indicated no evidence of violation of the parallel trend assumption for these DID models (eTable 4 in Supplement 1).

To explore potential differences in the experiences of uninsured individuals in states that did or did not expand Medicaid, as uninsured individuals from Medicaid expansion states may immediately qualify for Medicaid after Medicaid expansion, we stratified the analysis by Medicaid expansion status in another sensitivity analysis. We also conducted a sensitivity analysis including receipt of any cancer treatment within the follow-up period as a covariate to account for the possibility that some patients may not have received any treatment and therefore were unable to benefit from newer therapies. We used SAS, version 9.4 (SAS Institute Inc) for data preparation and descriptive statistics and R Studio, version 1.1.419 (R Project for Statistical Computing) for flexible parametric survival models (rstpm2 package). Statistical tests were 2-sided, and α was set at .05.

## Results

Among the 183 440 individuals included, the mean (SD) age was 55.5 (7.0) years; 43.5% were female, and 56.5% were male; and 4.7% were Hispanic, 13.4% were non-Hispanic Black, 77.0% were non-Hispanic White, and 4.2% were other race or ethnicity (Asian, Hispanic Black, Native American or Alaska Native, and multiple races) (Table 1). A total of 119 238 (65.0%) had private health insurance, 44 179 (24.1%) had Medicaid coverage, and 20 023 (10.9%) were uninsured at diagnosis. Compared with individuals with private health insurance, those with Medicaid or those without health insurance coverage were more likely to be younger, male, non-Hispanic Black or Hispanic, have more comorbid conditions, and be living in areas with lower family incomes or in nonmetropolitan areas. Across all included cancer sites, people with private health insurance at cancer diagnosis had better survival than people with Medicaid coverage or those without health insurance coverage both before and after the approval of ICIs (Figure; eFigure 2 in Supplement 1).

**Table 1.** Sample Characteristics for Patients With a Diagnosis of Stage IV Melanoma, NSCLC, or RCC in the NCDB

Characteristic	Patients, No. (%)			
	Total (N = 183 440)	Private (n = 119 238)	Medicaid (n = 44 179)	Uninsured (n = 20 023)
Cancer site				
Melanoma	12 048 (6.6)	8701 (7.3)	2056 (4.7)	1291 (6.4)
NSCLC	152 610 (83.2)	96 870 (81.2)	38 744 (87.7)	16 996 (84.9)
RCC	18 782 (10.2)	13 667 (11.5)	3379 (7.6)	1736 (8.7)
Age, y				
18-49	30 172 (16.4)	18 073 (15.2)	8354 (18.9)	3745 (18.7)
50-59	91 948 (50.1)	58 393 (49.0)	23 420 (53.0)	10 135 (50.6)
60-64	61 320 (33.4)	42 772 (35.9)	12 405 (28.1)	6143 (30.7)
Sex				
Female	79 799 (43.5)	53 539 (44.9)	18 494 (41.9)	7766 (38.8)
Male	103 641 (56.5)	65 699 (55.1)	25 685 (58.1)	12 257 (61.2)
Race and ethnicity				
Hispanic	8691 (4.7)	4113 (3.4)	2854 (6.5)	1724 (8.6)
Non-Hispanic Black	24 629 (13.4)	11 667 (9.8)	9344 (21.2)	3618 (18.1)
Non-Hispanic White	141 237 (77.0)	97 678 (81.9)	29 709 (67.2)	13 850 (69.2)
Other^a^	7728 (4.2)	5011 (4.2)	2026 (4.6)	691 (3.5)
Missing	1155 (0.6)	769 (0.6)	246 (0.6)	140 (0.7)
Zip code–level median income, as % of federal poverty level				
Low (≤138)	17 426 (9.5)	7613 (6.4)	7119 (16.1)	2694 (13.5)
Middle (139-400)	154 706 (84.3)	102 175 (85.7)	35 911 (81.3)	16 620 (83.0)
High (≥401)	10 348 (5.6)	8894 (7.5)	972 (2.2)	482 (2.4)
Missing	960 (0.5)	556 (0.5)	177 (0.4)	227 (1.1)
Metropolitan statistical area category of residence				
Metropolitan areas with population of ≥1 000 000	89 055 (48.5)	58 967 (49.5)	20 984 (47.5)	9104 (45.5)
Metropolitan areas with population <1 000 000	56 775 (31.0)	36 451 (30.6)	13 987 (31.7)	6337 (31.6)
Nonmetropolitan area	29 867 (16.3)	18 112 (15.2)	7944 (18.0)	3811 (19.0)
Missing	7743 (4.2)	5708 (4.8)	1264 (2.9)	771 (3.9)
Charlson-Deyo Comorbidity score				
0	127 792 (69.7)	85 746 (71.9)	28 279 (64.0)	13 767 (68.8)
1	38 177 (20.8)	23 472 (19.7)	10 334 (23.4)	4371 (21.8)
≥2	17 471 (9.5)	10 020 (8.4)	5566 (12.6)	1885 (9.4)
Facility type				
Community cancer program	13 104 (7.1)	7961 (6.7)	3672 (8.3)	1471 (7.3)
Comprehensive community cancer program	62 581 (34.1)	41 610 (34.9)	13 673 (30.9)	7298 (36.4)
Teach or research	42 275 (23.0)	24 180 (20.3)	12 380 (28.0.)	5715 (28.5)
National Cancer Institute program or network	25 736 (14.0)	19 037 (16.0)	5144 (11.6)	1555 (7.8)
Other and unknown	39 744 (21.7)	26 450 (22.2)	9310 (21.1)	3984 (19.9)
Time relative to the introduction of ICIs^b^				
Pre-ICI era	98 187 (53.5)	63 553 (53.3)	21 984 (49.8)	12 650 (63.2)
ICI era	85 253 (46.5)	55 685 (46.7)	22 195 (50.2)	7373 (36.8)
Receipt of any cancer treatment				
Yes	162 440 (88.6)	108 650 (91.1)	37 476 (84.8)	16 314 (81.5)
No	21 000 (11.5)	10 588 (8.9)	6703 (15.2)	3709 (18.5)

Abbreviations: ICI, immune checkpoint inhibitor; NCDB, National Cancer Database; NSCLC, non–small cell lung cancer; RCC, renal cell carcinoma.

^a^ Other included Asian, Hispanic Black, Native American or Alaska Native, and multiple races.

^b^ Immune checkpoint inhibitors were first approved on March 25, 2011, for stage IV melanoma; on March 4, 2015, for stage IV NSCLC; and on November 23, 2015, for stage IV RCC. To create balanced samples before and after the approval of ICIs, we identified patients who received a diagnosis at 18 to 64 years of age with stage IV melanoma between January 1, 2002, and December 31, 2019, and for stage IV NSCLC or stage IV RCC between January 1, 2010, and December 31, 2019. Patients who received a diagnosis before the introduction of ICIs were followed up until ICI approval. Patients who received a diagnosis before the introduction of ICIs were followed up until December 31, 2019.

**Figure.**
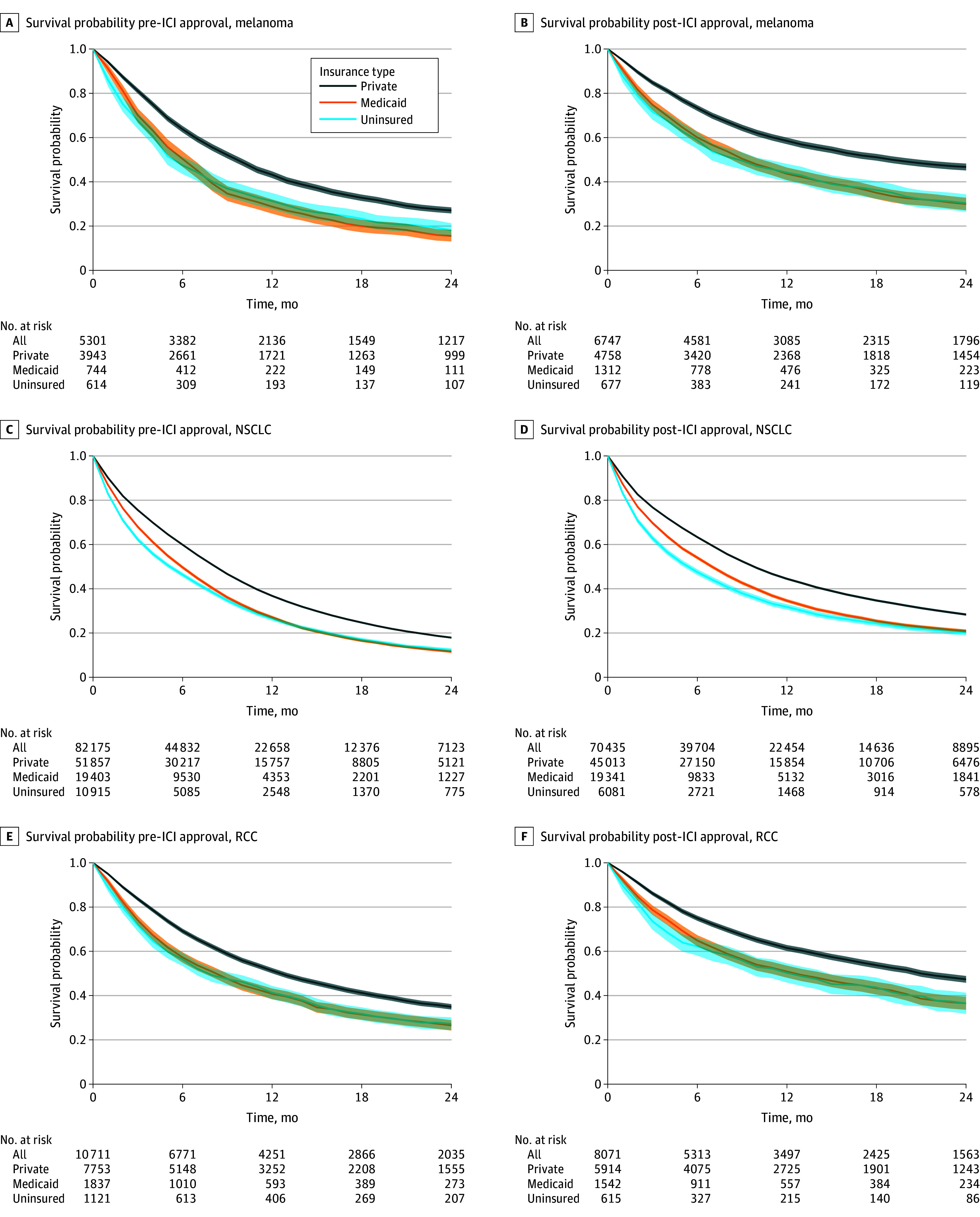
Kaplan-Meier Survival Curves Before and After US Food and Drug Administration Approval of Immune Checkpoint Inhibitors (ICIs) and Health Insurance Coverage Type ICIs were first approved on March 25, 2011, for stage IV melanoma; on March 4, 2015, for stage IV non–small cell lung cancer (NSCLC); and on November 23, 2015, for stage IV renal cell carcinoma (RCC). To create balanced samples before and after the approval of ICIs, we identified patients who received a diagnosis at 18 to 64 years of age for stage IV melanoma between January 1, 2002, and December 31, 2019, and for stage IV NSCLC or stage IV RCC between January 1, 2010, and December 31, 2019. Patients who received a diagnosis before the introduction of ICIs were followed up until ICI approval. Patients who received a diagnosis before the introduction of ICIs were followed up until December 31, 2019. The shaded areas indicate 95% CIs.

After the approval of ICIs, 2-year survival improved among patients for all selected cancers across all insurance types, with the exception of uninsured individuals with a diagnosis of RCC (Table 2). Among people with a diagnosis of stage IV melanoma, 2-year overall survival increased post-ICI approval among people who were uninsured (from 16.2% to 28.3%), those with Medicaid (from 14.1% to 29.6%), and those with private insurance (28.7% to 46.0%). After adjustment for sociodemographic characteristics, the survival disparity between those without health insurance and those with private insurance widened to 6.1 percentage points (pp) (95% CI, 1.7-10.6 pp) after the introduction of ICIs; improvements in survival were similar among individuals with Medicaid and privately insured individuals. Similarly, among individuals with a diagnosis of stage IV NSCLC, the survival disparity between those without insurance and those with private insurance widened to 1.3 pp (95% CI, 0.2-2.3 pp) after adjustment. Improvements in survival were similar between people with Medicaid and people with private insurance (DID for melanoma, −1.9 pp [95% CI, −5.6 to 1.8 pp]; DID for NSCLC, 0.4 pp [95% CI, −0.4 to 1.2 pp]; and DID for RCC, −3.8 pp [95% CI, −9.4 to 1.9 pp]). For individuals with a diagnosis of stage IV RCC, improvements in 2-year survival post-ICI approval were similar among uninsured, Medicaid, and privately insured groups (DID, −3.8 pp [95% CI, −9.4 to 1.9 pp] for Medicaid vs private insurance; DID, −5.8 pp [95% CI, −16.8 to 5.2 pp] for uninsured vs private insurance). After excluding data for the first year of ICI approval to account for lags in uptake, disparities in survival between uninsured and privately insured individuals widened for melanoma (DID, −5.0 pp [95% CI, −9.9 to −0.2 pp]), NSCLC (DID, −2.2 pp [95% CI, −3.4 to −1.0 pp]), and RCC (DID, −6.0 pp [95% CI, −11.5 to −0.6 pp]).

**Table 2.** Association of the Introduction of FDA-Approved ICIs and Disparities in 2-Year Survival Rate by Health Insurance Coverage Type After Diagnosis With Stage IV Cancer^a^

Characteristic	Survival rate, %		Difference (95% CI), %	Unadjusted		Adjusted^b^	
	Pre-ICI era	ICI era		DID, percentage points	*P* value	DID, percentage points	*P* value
**With first year data after ICI approval included**							
Melanoma							
Private health insurance	28.7	46.0	17.3 (13.0 to 21.6)	[Reference]	NA	[Reference]	NA
Medicaid	14.1	29.6	15.5 (12.1 to 18.8)	−1.8 (−5.7 to 2.1)	.36	−1.9 (−5.6 to 1.8)	.31
Uninsured	16.2	28.3	12.1 (7.8 to 16.4)	−5.2 (−10.0 to −0.4)	.03	−6.1 (−10.6 to −1.7)	.007
NSCLC							
Private health insurance	19.9	27.1	7.3 (6.3 to 8.3)	[Reference]	NA	[Reference]	NA
Medicaid	12.2	18.9	6.7 (6.0 to 7.3)	−0.6 (−1.5 to 0.3)	.17	0.4 (−0.4 to 1.2)	.32
Uninsured	11.0	15.2	4.2 (3.2 to 5.2)	−3.0 (−4.2 to −1.9)	<.001	−1.3 (−2.3 to −0.2)	.02
RCC							
Private health insurance	36.1	47.2	11.1 (6.8 to 15.5)	[Reference]	NA	[Reference]	NA
Medicaid	24.7	31.3	6.7 (3.5 to 9.8)	−4.5 (−8.1 to −0.9)	.02	−3.8 (−9.4 to 1.9)	.19
Uninsured	22.2	26.3	4.1 (−0.3 to 8.5)	−7.1 (−11.8 to −2.3)	.003	−5.8 (−16.8 to 5.2)	.30
**With first year data after ICI approval excluded**							
Melanoma							
Private health insurance	28.4	46.9	18.5 (13.9 to 23.1)	[Reference]	NA	[Reference]	NA
Medicaid	14.5	30.9	16.4 (13.0 to 19.8)	−2.1 (−6.1 to 1.9)	.30	−1.3 (−5.1 to 2.6)	.51
Uninsured	16.1	30.1	14.0 (9.4 to 18.5)	−4.5 (−9.5 to 0.5)	.08	−5.0 (−9.9 to −0.2)	.04
NSCLC							
Private health insurance	19.8	28.2	8.4 (7.3 to 9.5)	[Reference]	NA	[Reference]	NA
Medicaid	12.1	19.8	7.7 (6.9 to 8.5)	−0.7 (−1.7 to 0.3)	.15	0.4 (−0.6 to 1.3)	.45
Uninsured	10.9	15.4	4.5 (3.4 to 5.7)	−3.9 (−5.2 to −2.6)	<.001	−2.2 (−3.4 to −1.0)	<.001
RCC							
Private health insurance	35.9	48.5	12.6 (7.4 to 17.9)	[Reference]	NA	[Reference]	NA
Medicaid	24.5	32.2	7.8 (4.2 to 11.3)	−4.9 (−9.0 to −0.8)	.02	−4.5 (−9.5 to 0.6)	.08
Uninsured	22.0	27.1	5.1 (−0.2 to 10.3)	−7.6 (−13.2 to −2.0)	.008	−6.0 (−11.5 to −0.6)	.03

Abbreviations: DID, difference-in-differences; ICI, immune checkpoint inhibitor; NA, not applicable; NSCLC, non–small cell lung cancer; RCC, renal cell carcinoma.

^a^ Immune checkpoint inhibitors were first approved on March 25, 2011, for stage IV melanoma; on March 4, 2015, for stage IV NSCLC; and on November 23, 2015, for stage IV RCC. To create balanced samples before and after the approval of ICIs, we identified patients who received a diagnosis at 18 to 64 years of age with stage IV melanoma between January 1, 2002, and December 31, 2019, and for stage IV NSCLC or stage IV RCC between January 1, 2010, and December 31, 2019. Patients who received a diagnosis before the introduction of ICIs were followed up until ICI approval. Patients who received a diagnosis before the introduction of ICIs were followed up until December 31, 2019. A 6-group propensity score weighting approach was used in both unadjusted and adjusted DID models.

^b^ Models adjusted for age group, sex, race and ethnicity, zip code–level median income as percentage of federal poverty level, rural-urban status, Charlson-Deyo Comorbidity Index, facility type, state, and year of cancer diagnosis. The DID estimates reflect the changes in survival outcomes for uninsured individuals relative to individuals with private insurance, before and after the approval of ICIs. A negative DID estimate indicates that the uninsured group experienced a smaller improvement in survival compared with the privately insured group after ICI approval, indicating greater disparity by health insurance after the introduction of ICIs.

### Sensitivity Analyses

Similar improvement in survival were observed after ICI approval among patients with Medicaid and those with private insurance in this sensitivity analysis. When stratifying the analysis by Medicaid expansion status, no consistent or substantial differences by Medicaid expansion status across all cancer sites were found (eTable 5 in Supplement 1). The findings were largely unchanged after including receipt of any treatment as a covariate in the models (eTable 6 in Supplement 1). Also, the disparities in survival between uninsured patients and those with Medicaid widened for NSCLC (DID, 1.7 pp [95% CI, 0.6-2.8 pp]) (eTable 7 in Supplement 1).

## Discussion

Using recent nationwide cancer registry data, we found that the introduction of ICIs was associated with improved survival for patients with a new diagnosis of advanced-stage cancers across all insurance types. However, the survival gap between privately insured and uninsured individuals with a new diagnosis of stage IV melanoma or NSCLC widened significantly. After excluding data for the first year of ICI approval to account for lags in uptake, the disparities in survival between uninsured and privately insured patients widened for melanoma, NSCLC, and RCC. Similar improvements in survival among individuals with Medicaid and privately insured individuals were also observed.

Our findings have significant implications in the context of longstanding disparities in cancer outcomes in the US, where individuals with lower socioeconomic status or without health insurance have experienced worse survival rates.^15^ This study is timely given the increasing use of ICIs for many cancers in both early and late stages of diagnosis and for first-line treatment, as well as subsequent lines of treatment. In addition, the evolving landscape of ICIs, with new approvals and combination therapies, could result in further widening of disparities in outcomes by health insurance coverage.

Our analyses extend previous research on the widening of preexisting disparities by health insurance coverage associated with the introduction of ICIs for multiple cancer types. For example, studies undertaken immediately after the first FDA approval of ICIs in 2011 found that individuals with a new diagnosis of melanoma who were uninsured were less likely to receive immunotherapy compared with those with private health insurance coverage.^29,30^ Another study, using SEER-18 registry data from 14 states, found that overall survival improved after FDA approval of ICIs among individuals with a diagnosis of stage IV melanoma with health insurance coverage, but no improvement was observed among individuals without health insurance.^31^ However, these studies did not include data from all states, they did not examine NSCLC and RCC, and they did not use a DID study design to analyze the changes in disparities. The widening of disparities after excluding the first year after approval for RCC could be associated with slower uptake of ICIs among patients with RCC.

Our findings that the introduction of ICIs was associated with increased disparities between uninsured and privately insured patients have important policy implications. Studies have shown that several federal and state policies under the ACA, such as Medicaid expansion, which expands Medicaid eligibility to individuals with incomes 138% or less of the federal poverty level,^32^ and the establishment of the health insurance marketplace, which provides premium and cost-sharing subsidies to low- and middle-income families, were associated with increases in health insurance coverage.^33^ Our findings that the magnitude of improvement in survival after FDA approval of ICIs was similar among people with private health insurance and Medicaid coverage for multiple cancers suggest that expanding Medicaid to individuals without health insurance coverage may improve their access to ICI treatment. However, as of May 2025, 10 states had not adopted Medicaid expansion. Most of the nonexpansion states also have a lower median family income and a higher cancer mortality burden compared with expansion states, which may lead to even greater cancer disparities by geographic region and socioeconomic status in the future. That said, we did find similar survival improvements among individuals with Medicaid and individuals with private insurance, which may reflect Medicaid’s required coverage of FDA-approved oncology therapies, including ICIs.^34^ Nevertheless, differences in clinician access, prior authorization, and state-level variation in Medicaid reimbursement and coverage policies may influence treatment experiences and warrant further research.

In addition to improving health insurance coverage, efforts to provide comprehensive financial assistance and patient navigation programs to uninsured patients could improve access to new treatments, such as ICIs, and reduce disparities by health insurance coverage. Some cancer centers and pharmaceutical companies provide financial assistance programs to support ICI treatment access for uninsured patients and those with lower incomes. However, even with these programs, patients may experience delays in ICI treatment initiation due to uncertainties during the application process and lack of clinician awareness. Meanwhile, even with financial assistance, many patients may still face high out-of-pocket spending for their ICIs and related cancer treatments.^35^ Thus, providing comprehensive financial assistance programs and patient navigation are warranted.

### Limitations

This study has some limitations; one is that health insurance was measured only at the time of cancer diagnosis, and information on health insurance coverage changes was not available. Some individuals who were initially uninsured may have become eligible for Medicaid or obtained other coverage after diagnosis, while some patients with private health insurance may have no longer been able to work, thus possibly losing household income and their employer-based private health insurance. Future research should explore how insurance coverage continuity is associated with receipt of high-cost treatments, such as ICIs.

This study has other limitations. First, we examined only all-cause mortality because cancer-specific mortality information is not available in the NCDB. However, additional analyses we conducted comparing overall and cancer-specific survival using SEER data indicated that all-cause and cancer-specific mortality were generally similar over our study period in a similar sample of individuals with a new diagnosis of stage IV disease. Second, we focused on only 3 cancer sites because for cancers for which ICIs were approved after 2016, available postapproval sample sizes are currently inadequate. Also, there is insufficient information in the NCDB on subtype for some cancers. In addition, within the time frame of our analyses, there might have been differences in the receipt of other new and effective cancer treatments, as well as changes in epidemiologic patterns (eg, smoking) by health insurance coverage, although none of these changes would have been unobservable in our data. Moreover, lack of information on specific treatment agents in the NCDB limited our ability to determine the percentage of patients receiving ICIs, which restricts our analysis to population-level effects rather than direct treatment effects.

## Conclusions

In this cross-sectional study using nationwide cancer registry data, we found that the introduction of ICIs was associated with improved survival across all insurance types for individuals with a new diagnosis of advanced-stage melanoma, NSCLC, or RCC. However, the introduction of ICIs was associated with a widening of preexisting survival disparities between privately insured and uninsured individuals. This gap is likely due to barriers to accessing ICI treatment; programs that reduce barriers to care, such as expanding access to health insurance coverage, providing comprehensive financial assistance to people without health insurance coverage, and making new treatments more affordable, may help to mitigate these disparities.

## Notes

Supplement 1. **eTable 1.** List of Immune Checkpoint Inhibitors Approved by the FDA **eTable 2.** Excluded Patients due to Missing Data, by Health Insurance Coverage **eTable 3.** Overall and Cancer-Specific Two-Year Survival Rate for Stage IV Selected Cancers, SEER 22 **eTable 4.** Parallel Trend Assumption Tests **eTable 5.** Association of the Introduction of FDA-Approved Immune Checkpoint Inhibitors and Disparities in Two-Year Survival by Health Insurance Coverage Following Diagnosis With Cancer, by Medicaid Expansion Status **eTable 6.** Association of the Introduction of FDA-Approved Immune Checkpoint Inhibitors and Disparities in Two-Year Survival by Health Insurance Coverage Type Following Diagnosis With Stage IV Cancer **eTable 7.** Association of the Introduction of FDA-Approved Immune Checkpoint Inhibitors and Disparities in Two-Year Survival Comparing Individuals Uninsured and With Medicaid Coverage Following Diagnosis With Stage IV Cancer **eFigure 1.** Sample Selection **eFigure 2.** Kaplan-Meier Survival Curves by Pre- and Post-FDA ICI Approval Period and Health Insurance Coverage Type for Patients Diagnosed With Stage IV Melanoma, Non-Small Cell Lung Cancer, and Renal Cell Carcinoma
eMethods.

Supplement 2.
Data Sharing Statement
