# Supplementary material for: Immune Checkpoint Inhibitors and Survival Disparities by Health Insurance Coverage Among Patients With Metastatic Cancer
Source: JAMA Netw Open. 2025 Jul 7;8(7):e2519274. doi: 10.1001/jamanetworkopen.2025.19274 (PMC12235497; doi:10.1001/jamanetworkopen.2025.19274)
Supplement: Supplement 1. — eTable 1. List of Immune Checkpoint Inhibitors Approved by the FDA eTable 2. Excluded Patients due to Missing Data, by Health Insurance Coverage eTable 3. Overall and Cancer-Specific Two-Year Survival Rate for Stage IV Selected Cancers, SEER 22 eTable 4. Parallel Trend Assumption Tests eTable 5. Association of the Introduction of FDA-Approved Immune Checkpoint Inhibitors and Disparities in Two-Year Survival by Health Insurance Coverage Following Diagnosis With Cancer, by Medicaid Expansion Status eTable 6. Association of the Introduction of FDA-Approved Immune Checkpoint Inhibitors and Disparities in Two-Year Survival by Health Insurance Coverage Type Following Diagnosis With Stage IV Cancer eTable 7. Association of the Introduction of FDA-Approved Immune Checkpoint Inhibitors and Disparities in Two-Year Survival Comparing Individuals Uninsured and With Medicaid Coverage Following Diagnosis With Stage IV Cancer eFigure 1. Sample Selection eFigure 2. Kaplan-Meier Survival Curves by Pre- and Post-FDA ICI Approval Period and Health Insurance Coverage Type for Patients Diagnosed With Stage IV Melanoma, Non-Small Cell Lung Cancer, and Renal Cell Carcinoma eMethods. [file jamanetwopen-e2519274-s001.pdf]

Supplementary Online Content

Zhao J, Graetz I, Howard D, Yabroff KR, Lipscomb J. Immune checkpoint inhibitors and survival disparities by health insurance coverage among patients with metastatic cancer. *JAMA Netw Open.* 2025;8(7):e2519274. doi:10.1001/jamanetworkopen.2025.19274

eTable 1. List of Immune Checkpoint Inhibitors Approved by the FDA

eTable 2. Excluded Patients due to Missing Data, by Health Insurance Coverage

eTable 3. Overall and Cancer-Specific Two-Year Survival Rate for Stage IV Selected Cancers, SEER 22

eTable 4. Parallel Trend Assumption Tests

eTable 5. Association of the Introduction of FDA-Approved Immune Checkpoint Inhibitors and Disparities in Two-Year Survival by Health Insurance Coverage Following Diagnosis With Cancer, by Medicaid Expansion Status

eTable 6. Association of the Introduction of FDA-Approved Immune Checkpoint Inhibitors and Disparities in Two-Year Survival by Health Insurance Coverage Type Following Diagnosis With Stage IV Cancer

eTable 7. Association of the Introduction of FDA-Approved Immune Checkpoint Inhibitors and Disparities in Two-Year Survival Comparing Individuals Uninsured and With Medicaid Coverage Following Diagnosis With Stage IV Cancer

eFigure 1. Sample Selection

eFigure 2. Kaplan-Meier Survival Curves by Pre- and Post-FDA ICI Approval Period and Health Insurance Coverage Type for Patients Diagnosed With Stage IV Melanoma, Non-Small Cell Lung Cancer, and Renal Cell Carcinoma

eMethods.

This supplementary material has been provided by the authors to give readers additional information about their work.

© 2025 Zhao J et al. *JAMA Network Open.*

eTable 1. List of Immune Checkpoint Inhibitors Approved by the FDA

	Time ICI first approved	ICI agent first approved	Other ICI agents approved by 2019
Melanoma	3/25/2011	Ipilimumab (CTLA-4)	Nivolumab (PD-1), Pembrolizumab (PD-1)
Non-small cell lung cancer	3/4/2015	Nivolumab (PD-1)	Pembrolizumab (PD-1), Atezolizumab (PD-1), Durvalumab (PD-L1)
Renal cell carcinoma	11/23/2015	Nivolumab (PD-1)	Pembrolizumab (PD-1), Atezolizumab (PD-1)

© 2025 Zhao J et al. *JAMA Network Open.*

eTable 2. Excluded Patients due to Missing Data, by Health Insurance Coverage

	Included in the sample	Excluded due to missing	P-value
	n (%)	n (%)	
Melanoma			
Private health insurance	8701 (98.6)	121 (1.4)	0.018
Medicaid	2056 (97.8)	46 (2.2)	
Uninsured	1291 (98.2)	24 (1.8)	
Non-small cell lung cancer			
Private health insurance	96870 (99.4)	619 (0.6)	<.0001
Medicaid	38744 (98.9)	414 (1.1)	
Uninsured	16996 (99.1)	153 (0.9)	
Renal cell carcinoma			
Private health insurance	13667 (98.9)	148 (1.1)	0.4894
Medicaid	3379 (98.8)	41 (1.2)	
Uninsured	1736 (98.6)	24 (1.4)	

***** P-value compares percentage of missing by health insurance coverage

© 2025 Zhao J et al. *JAMA Network Open.*

eTable 3. Overall and Cancer-Specific Two-Year Survival Rate for Stage IV Selected Cancers, SEER 22

	Overall survival rate and 95% confidence interval	Cancer-specific survival rate and 95% confidence interval
Melanoma (years 2002-2019)	34.9 (32.7 - 37.2)	36.5 (34.2 - 38.8)
Lung cancer (years 2010-2019)	15.6 (15.3 - 16.0)	16.9 (16.6 - 17.3)
Kidney and renal pelvis cancer (years 2010-2019)	30.7 (29.4 - 32.0)	33.1 (31.7 - 34.4)

© 2025 Zhao J et al. *JAMA Network Open.*

eTable 4. Parallel Trend Assumption Tests

	P-value for the interaction term of health insurance by quarter			
	Medicaid vs. Private		Uninsured vs. Private	
	Unadjusted	Adjusted	Unadjusted	Adjusted
Melanoma	0.056	0.166	0.873	0.689
Non-small cell lung cancer	0.228	0.368	0.106	0.151
Renal cell carcinoma	0.826	0.479	0.677	0.733

* Models adjusted for age group, sex (except for breast cancer), race and ethnicity, zip-code level median income as percent of FPL, rural-urban status, Charlson-Deyo comorbidity index, facility type, state, and year of cancer diagnosis. A six-group propensity score weighting approach was in DID models. P-value> 0.05 indicates that the parallel trend assumption is not violated.

© 2025 Zhao J et al. *JAMA Network Open.*

eTable 5. Association of the Introduction of FDA-Approved Immune Checkpoint Inhibitors and Disparities in Two-Year Survival by Health Insurance Coverage Following Diagnosis With Cancer, by Medicaid Expansion Status

	n	Pre-ICI	ICI	Difference	DID	P-value	Adjusted DID [l]	P-value
**Melanoma**								
**State expanded Medicaid in 2014**								
Private health insurance	4526	29.04	45.13	16.09 (5.7to26.48)				
Medicaid	1176	15.84	24.62	8.78 (3.03to14.52)	-7.31 (-14.04to-0.59)	0.033	-5.09 (-11.53to1.35)	0.121
Uninsured	430	17.43	26.00	8.57 (-1.82to18.96)	-7.52 (-18.48to3.44)	0.179	-6.48 (-17.28to4.32)	0.239
**Late expansion states**								
Private health insurance	1211	31.50	40.18	8.67 (-5.14to22.49)				
Medicaid	237	10.41	26.91	16.49 (5.16to27.82)	7.82 (-5.42to21.06)	0.313	7.07 (-5.86to20.01)	0.284
Uninsured	143	19.02	17.40	-1.62 (-15.43to12.2)	-10.29 (-25.71to5.13)	0.191	-8.04 (-23.62to7.54)	0.312
**Non expansion states**								
Private health insurance	2964	26.67	49.60	22.93 (16.1to29.76)				
Medicaid	643	16.04	30.98	14.94 (7.62to22.27)	-7.98 (-16.38to0.41)	0.062	-5.89 (-14.04to2.25)	0.156
Uninsured	718	12.96	32.97	20.01 (13.18to26.84)	-2.91 (-10.88to5.06)	0.474	-2.1 (-9.9to5.7)	0.597
**Non-small cell lung cancer**								
**State expanded Medicaid in 2014**								
Private health insurance	49938	20.99	29.20	8.21 (5.83to10.6)				
Medicaid	21212	13.67	21.07	7.4 (6.34to8.45)	-0.82 (-2.17to0.54)	0.238	0.21 (-1.05to1.47)	0.743
Uninsured	4970	11.19	16.85	5.66 (3.27to8.04)	-2.55 (-5.09to-0.02)	0.048	-1.87 (-4.07to0.33)	0.096
**Late expansion states**								
Private health insurance	14953	17.28	25.13	7.85 (4.72to10.99)				
Medicaid	5012	9.95	17.48	7.53 (5.55to9.51)	-0.32 (-2.76to2.12)	0.796	0.18 (-2.1to2.46)	0.877
Uninsured	2258	10.17	15.96	5.79 (2.65to8.92)	-2.06 (-5.51to1.38)	0.240	-1.03 (-4.17to2.11)	0.520
**Non expansion states**								
Private health insurance	31979	18.90	27.80	8.9 (7.48to10.33)				
Medicaid	12520	10.50	18.17	7.68 (6.37to8.99)	-1.23 (-2.89to0.43)	0.148	0.53 (-1.05to2.1)	0.512
Uninsured	9768	10.72	14.40	3.68 (2.26to5.1)	-5.23 (-6.97to-3.48)	<0.001	-2.97 (-4.61to-1.33)	<0.001
**Renal cell carcinoma**								
**State expanded Medicaid in 2014**								
Private health insurance	6894	34.41	47.80	13.39 (5.86to20.93)				
Medicaid	2027	26.75	37.60	10.85 (5.41to16.29)	-2.54 (-8.68to3.6)	0.417	-2.7 (-8.7to3.3)	0.378
Uninsured	537	21.55	24.97	3.42 (-4.11to10.95)	-9.97 (-18.03to-1.92)	0.015	-8.28 (-16.18to-0.39)	0.040
**Late expansion states**								
Private health insurance	1956	35.32	51.29	15.97 (0.21to31.72)				
Medicaid	363	23.03	30.24	7.22 (-3.13to17.56)	-8.75 (-20.38to2.88)	0.140	-8.72 (-20.16to2.72)	0.135
Uninsured	183	19.46	28.66	9.19 (-6.56to24.95)	-6.77 (-23.4to9.85)	0.425	-4.97 (-20.95to11.01)	0.542
**Non expansion states**								
Private health insurance	4817	36.22	48.55	12.32 (2.82to21.83)				
Medicaid	989	22.20	30.00	7.8 (1.67to13.94)	-4.52 (-11.58to2.53)	0.209	-2.89 (-9.77to3.98)	0.409
Uninsured	1016	25.05	32.30	7.25 (-2.26to16.75)	-5.08 (-15.2to5.04)	0.325	-3.82 (-13.62to5.97)	0.444

© 2025 Zhao J et al. *JAMA Network Open.*

* Immune checkpoint inhibitors (ICIs) were first approved for stage IV melanoma on 03/25/2011), for stage IV non-small cell lung cancer (NSCLC) on 03/04/2015, and for stage IV renal cell carcinoma (RCC) on 11/23/2015. To create balanced samples before and after the approval of ICI, we identified patients diagnosed at age 18-64 years with stage IV melanoma between January 1, 2002 and December 31, 2019, and for stage IV NSCLC, and stage IV renal cell carcinoma between January 1, 2010 and December 31, 2019. Patients diagnosed before the introduction of ICI were followed up till the ICI approval. Patients diagnosed before the introduction of ICI were followed up till December 31st, 2019. A six-group propensity score weighting approach was in both unadjusted and adjusted difference-in-differences (DID) models. States expanded Medicaid in 2014 included: Arizona, Arkansas, California, Colorado, Connecticut, Delaware, District of Columbia, Hawaii, Illinois, Iowa, Kentucky, Maryland, Massachusetts, Minnesota, Nevada, New Jersey, New Mexico, New York, North Dakota, Ohio, Oregon, Rhode Island, Vermont, Washington, West Virginia, Michigan, and New Hampshire. Late expansion states included Alaska, Indiana, Louisiana, Maine, Montana, Pennsylvania, and Virginia. Non expansion states included Alabama, Florida, Georgia, Idaho, Kansas, Mississippi, Missouri, Nebraska, North Carolina, Oklahoma, South Carolina, South Dakota, Tennessee, Texas, Utah, Wisconsin, and Wyoming.

‖ Models adjusted for age group, sex, race and ethnicity, zip-code level median income as percent of federal poverty line (FPL), rural-urban status, Charlson-Deyo comorbidity index, facility type, state, and year of cancer diagnosis. The DID estimates reflect the changes in survival outcomes for uninsured individuals relative to individuals with private insurance, before and after the approval ICIs. A negative sign in the DID estimate indicates that the uninsured group experienced a smaller improvement in survival compared to the privately insured group following ICI approval, indicating greater disparity by health insurance after the introduction of ICIs.

© 2025 Zhao J et al. *JAMA Network Open.*

eTable 6. Association of the Introduction of FDA-Approved Immune Checkpoint Inhibitors and Disparities in Two-Year Survival by Health Insurance Coverage Type Following Diagnosis With Stage IV Cancer*

	Pre-ICI	ICI	Difference	DID	P-value	Adjusted DID[‖]	P-value
**Melanoma**							
Private health insurance	28.69	45.97	17.29(12.97to21.6)				
Medicaid	14.14	29.60	15.46(12.12to18.81)	-1.82(-5.74to2.09)	0.361	-0.56(-4.2to3.07)	0.761
Uninsured	16.18	28.26	12.08(7.77to16.39)	-5.21(-9.97to-0.44)	0.032	-4.8(-9.24to-0.35)	0.034
**Non-small cell lung cancer**							
Private health insurance	19.87	27.13	7.26(6.25to8.26)				
Medicaid	12.20	18.85	6.65(5.97to7.33)	-0.61(-1.47to0.26)	0.170	-0.19(-0.97to0.6)	0.643
Uninsured	10.96	15.20	4.24(3.23to5.24)	-3.02(-4.16to-1.89)	<0.001	-1.78(-2.84to-0.72)	0.001
**Renal cell carcinoma**							
Private health insurance	36.05	47.19	11.14(6.77to15.52)				
Medicaid	24.67	31.34	6.67(3.49to9.84)	-4.48(-8.11to-0.85)	0.016	-4.18(-10.3to1.93)	0.180
Uninsured	22.19	26.28	4.09(-0.28to8.46)	-7.05(-11.77to-2.34)	0.003	-4.35(-11.24to2.53)	0.215

* Immune checkpoint inhibitors (ICIs) were first approved for stage IV melanoma on 03/25/2011), for stage IV non-small cell lung cancer (NSCLC) on 03/04/2015, and for stage IV renal cell carcinoma (RCC) on 11/23/2015. To create balanced samples before and after the approval of ICI, we identified patients diagnosed at age 18-64 years with stage IV melanoma between January 1, 2002 and December 31, 2019, and for stage IV NSCLC, and stage IV renal cell carcinoma between January 1, 2010 and December 31, 2019. Patients diagnosed before the introduction of ICI were followed up till the ICI approval. Patients diagnosed before the introduction of ICI were followed up till December 31st, 2019. A six-group propensity score weighting approach was in both unadjusted and adjusted difference-in-differences (DID) models.

‖ Models adjusted for age group, sex, race and ethnicity, zip-code level median income as percent of federal poverty line (FPL), rural-urban status, Charlson-Deyo comorbidity index, facility type, state, receipt of any treatment, and year of cancer diagnosis. The DID estimates reflect the changes in survival outcomes for uninsured individuals relative to individuals with private insurance, before and after the approval ICIs. A negative sign in the DID estimate indicates that the uninsured group experienced a smaller improvement in survival compared to the privately insured group following ICI approval, indicating greater disparity by health insurance after the introduction of ICIs.

© 2025 Zhao J et al. *JAMA Network Open.*

eTable 7. Association of the Introduction of FDA-Approved Immune Checkpoint Inhibitors and Disparities in Two-Year Survival Comparing Individuals Uninsured and With Medicaid Coverage Following Diagnosis With Stage IV Cancer*

	Pre-ICI	ICI	Difference	DID	P-value	Adjusted DID‖	P-value
**Melanoma**							
Medicaid	14.14	29.60	15.46(12.12to18.81)				
Uninsured	16.18	28.26	12.08(7.77to16.39)	-3.38(--8.84to2.07)	0.224	-4.80 (-10.04to0.04)	0.073
**Non-small cell lung cancer**							
Medicaid	12.20	18.85	6.65 (5.97to7.33)				
Uninsured	10.96	15.20	4.24 (3.23to5.24)	-2.42 (-3.63to-1.20)	<0.001	-1.66 (-2.77to-0.56)	0.032
**Renal cell carcinoma**							
Medicaid	24.67	31.34	6.67 (3.49to9.84)				
Uninsured	22.19	26.28	4.09 (-0.28to8.46)	-2.58 (-7.98to-2.82)	0.350	-2.01 (-9.69to5.66)	0.607

* Immune checkpoint inhibitors (ICIs) were first approved for stage IV melanoma on 03/25/2011), for stage IV non-small cell lung cancer (NSCLC) on 03/04/2015, and for stage IV renal cell carcinoma (RCC) on 11/23/2015. To create balanced samples before and after the approval of ICI, we identified patients diagnosed at age 18-64 years with stage IV melanoma between January 1, 2002 and December 31, 2019, and for stage IV NSCLC, and stage IV renal cell carcinoma between January 1, 2010 and December 31, 2019. Patients diagnosed before the introduction of ICI were followed up till the ICI approval. Patients diagnosed before the introduction of ICI were followed up till December 31st, 2019. A six-group propensity score weighting approach was in both unadjusted and adjusted difference-in-differences (DID) models.

‖ Models adjusted for age group, sex, race and ethnicity, zip-code level median income as percent of federal poverty line (FPL), rural-urban status, Charlson-Deyo comorbidity index, facility type, state, and year of cancer diagnosis. The DID estimates reflect the changes in survival outcomes for uninsured individuals relative to individuals with private insurance, before and after the approval ICIs. A negative sign in the DID estimate indicates that the uninsured group experienced a smaller improvement in survival compared to the privately insured group following ICI approval, indicating greater disparity by health insurance after the introduction of ICIs.

© 2025 Zhao J et al. *JAMA Network Open.*

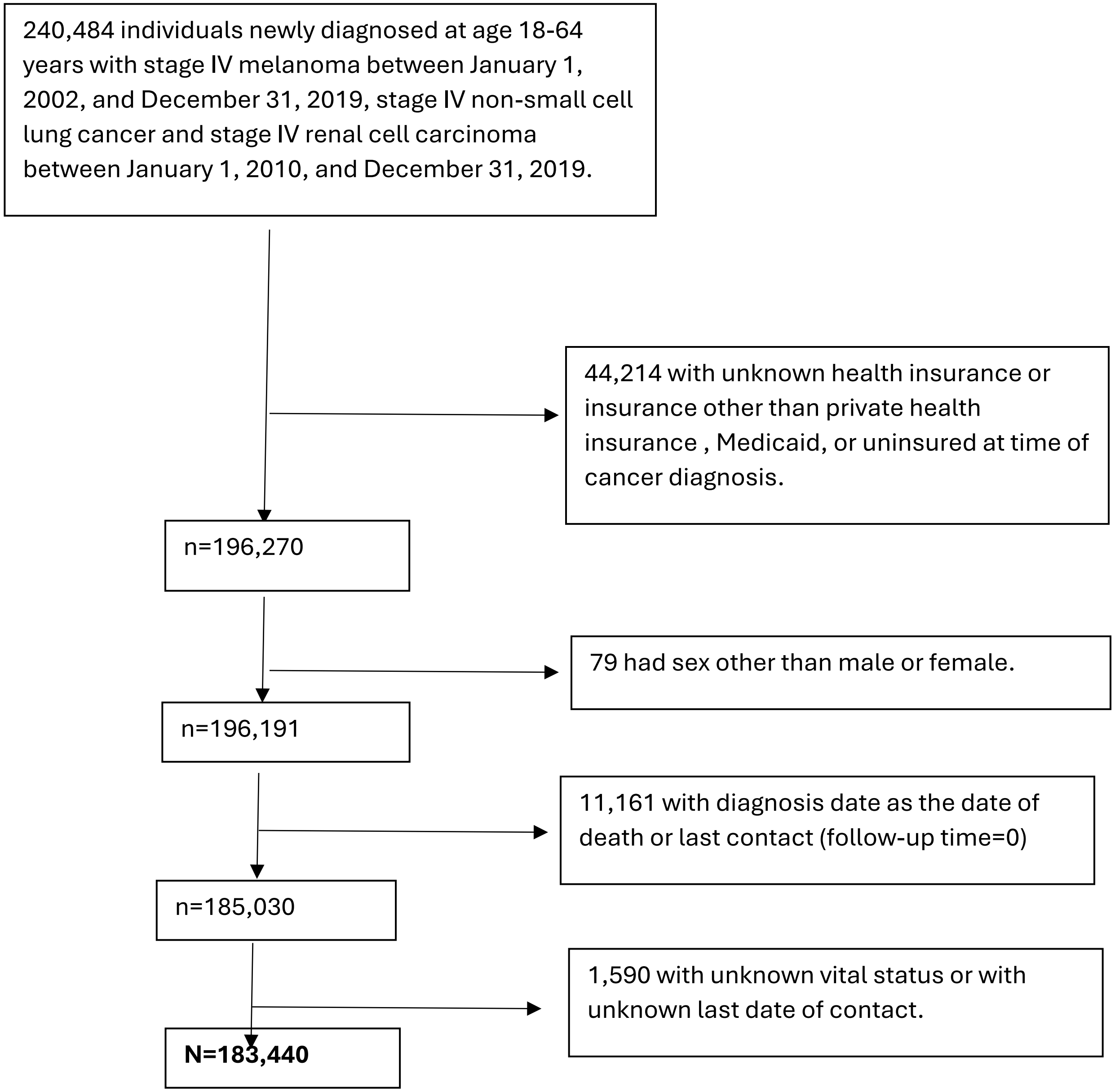

eFigure 1. Sample Selection

© 2025 Zhao J et al. *JAMA Network Open.*

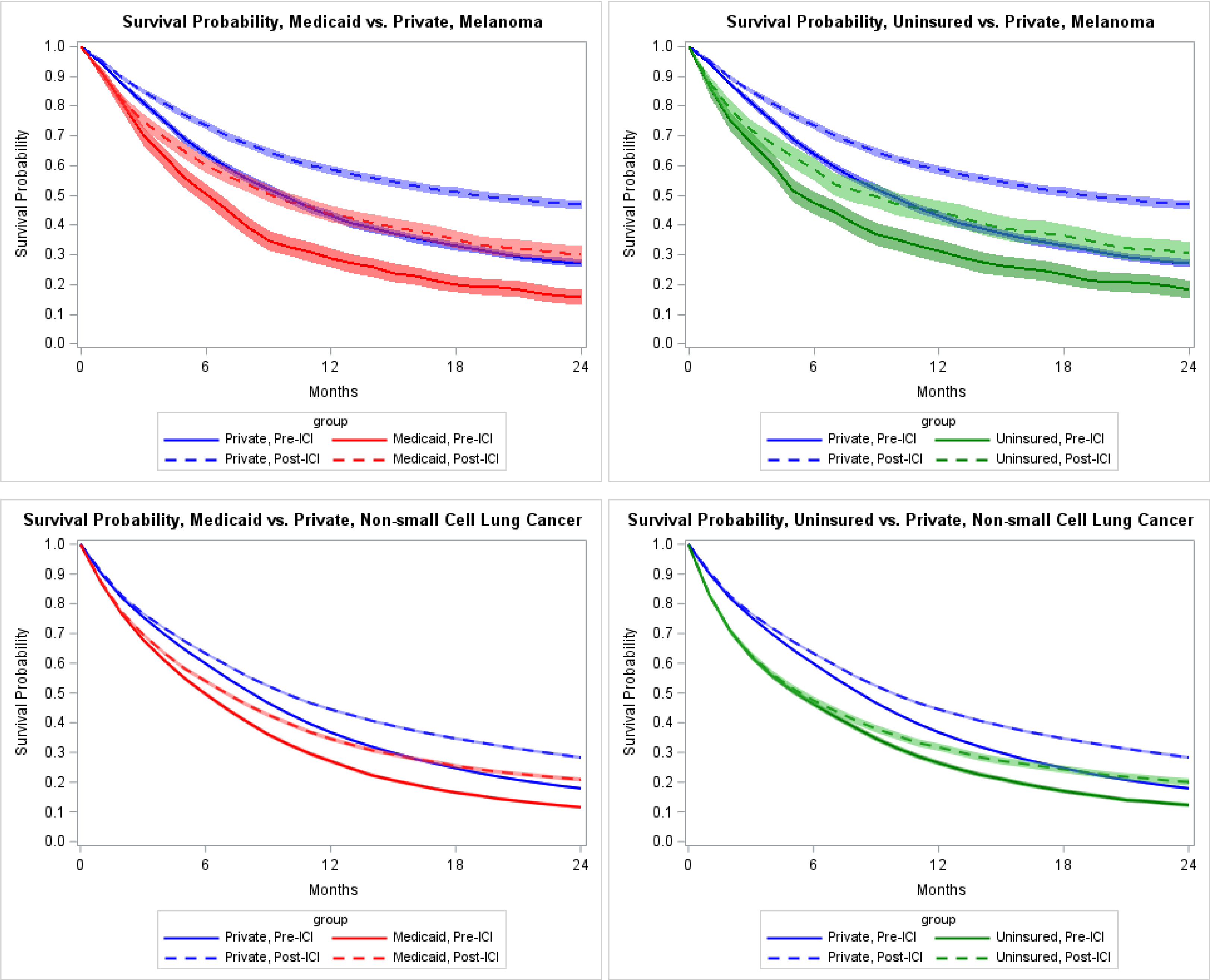

© 2025 Zhao J et al. *JAMA Network Open.*

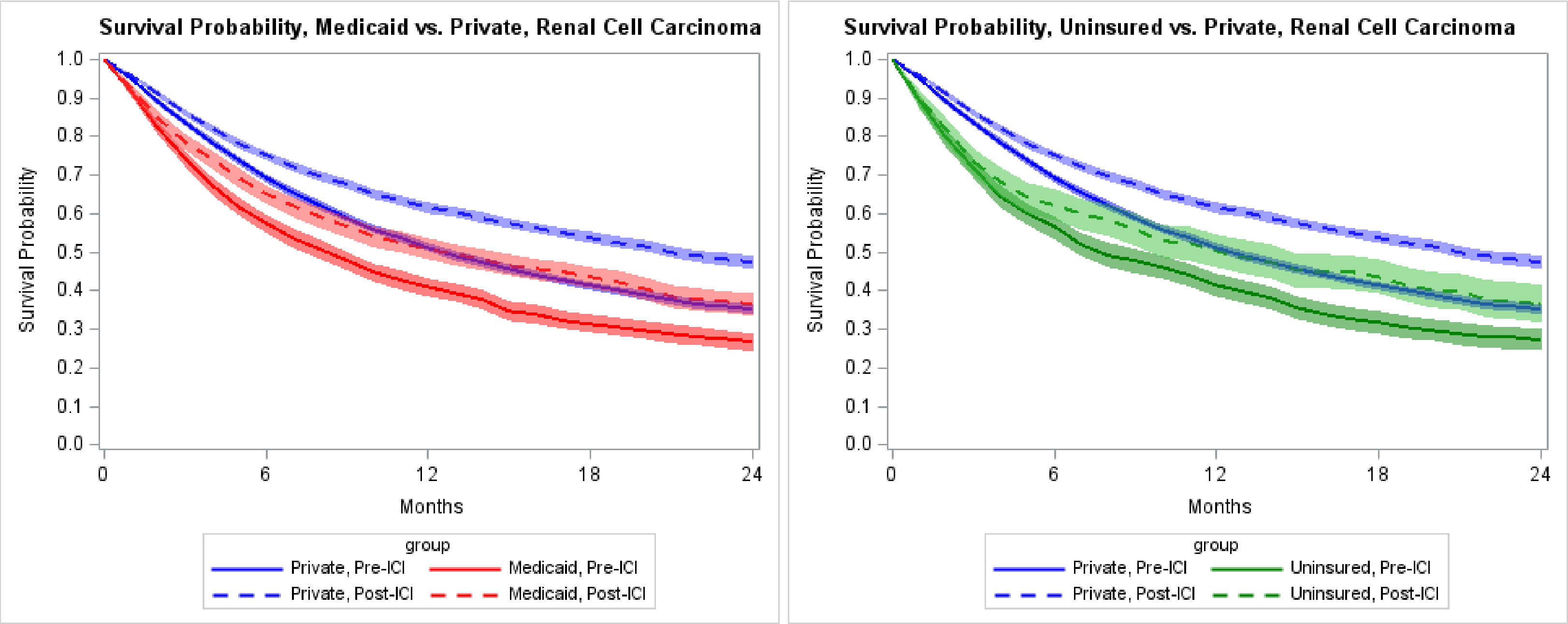

eFigure 2. Kaplan-Meier Survival Curves by Pre- and Post-FDA ICI Approval Period and Health Insurance Coverage Type for Patients Diagnosed With Stage IV Melanoma, Non-Small Cell Lung Cancer, and Renal Cell Carcinoma

Immune checkpoint inhibitors (ICIs) were first approved for stage IV melanoma on 03/25/2011), for stage IV non-small cell lung cancer (NSCLC) on 03/04/2015, and for stage IV renal cell carcinoma (RCC) on 11/23/2015. To create balanced samples before and after the approval of ICI, we identified patients diagnosed at age 18-64 years with stage IV melanoma between January 1, 2002 and December 31, 2019, and for stage IV NSCLC, and stage IV renal cell carcinoma between January 1, 2010 and December 31, 2019. Patients diagnosed before the introduction of ICI were followed up till the ICI approval. Patients diagnosed before the introduction of ICI were followed up till December 31st, 2019.

© 2025 Zhao J et al. *JAMA Network Open.*

eMethods.

Six-group propensity score weighting in DID model

Difference-in-difference (DID) approach is a commonly used approach to study the effects of policies or programs. However, disproportional changes in sample composition in treatment and control group overtime may introduce bias in the DID study. In this study, Medicaid expansion changed the sample composition by health insurance over time. To account for potential bias introduced by changes in sample composition by health insurance due to Medicaid expansion, we used a six-group (private pre-ICI (group 1), private post-ICI (group 2), Medicaid pre-ICI (group 3), Medicaid post-ICI (group 4), uninsured pre-ICI (group 5), uninsured post-ICI (group 6)) propensity score weighting approach in DID models.

In particular, the propensity score is defined as the probability of being in Group 1 (versus Groups 2, 3, 4, 5, or 6). We used multinomial logistic regression to estimate the propensity scores, conditional on age group, sex, race and ethnicity, zip-code level median income as a percent of FPL, rural-urban status, Charlson-Deyo comorbidity index, and facility type. These variables were selected as their compositions by health insurance were likely to change after Medicaid expansion. Hence, each individual will have six resulting propensity scores, $e_k(X_i)$, representing the probability of being in Group k, for k=1 to 6.

For individual i:

$$w_i=e_1(X_i)/e_g(X_i)$$

where g refers to the group that individual i was actually in.

Therefore, patients of Group 1 will be assigned a weight of 1, whereas patients in other groups will receive a weight proportional to the probability of their belonging to Group 1 compared to the probability of their actual group.

The inverse probability weights (IPW) is the inverse of the assigned weight. Additionally, we standardized the inverse probability weights (SIPW) by dividing the IPW by the average IPW within each specific group, to improve the balance between groups and enhance the validity of the comparisons.
