# Supplementary material for: Immune Checkpoint Inhibitors and Survival Disparities by Health Insurance Coverage Among Patients With Metastatic Cancer
Source: JAMA Netw Open. 2025 Jul 7;8(7):e2519274. doi: 10.1001/jamanetworkopen.2025.19274 (PMC12235497; doi:10.1001/jamanetworkopen.2025.19274)
Supplement: Supplement 2. — Data Sharing Statement [file jamanetwopen-e2519274-s002.pdf]

Data Sharing Statement

Zhao. Immune Checkpoint Inhibitors and Survival Disparities by Health Insurance Coverage Among Patients With Metastatic Cancer. *JAMA Netw Open*. Published July 07, 2025. doi:10.1001/jamanetworkopen.2025.19274

Data
Data available: No

Additional Information
Explanation for why data not available: Some data used for this study are not publicly available according to the Data Use Aggrement.
